# Psychometric validation of the Laval developmental benchmarks scale for family medicine

**DOI:** 10.1186/s12909-021-02797-3

**Published:** 2021-06-27

**Authors:** Jean-Sébastien Renaud, Miriam Lacasse, Luc Côté, Johanne Théorêt, Christian Rheault, Caroline Simard

**Affiliations:** 1 Department of Family and Emergency Medicine, Laval University, 1050, avenue de la Médecine, Université Laval, Québec, G1V 0A6 Canada; 2 Office of Education and Continuing Professional Development, Laval University, 1050, avenue de la Médecine, Université Laval, Québec, G1V 0A6 Canada; 3 Primary Care Research Centre affiliated with Laval University (CERSSPL-U, 1050, avenue de la Médecine, Université Laval, Québec, G1V 0A6 Canada; 4 Educational Leadership Chair in Health Professions Education CMA-MDM, 1050, avenue de la Médecine, Université Laval, Québec, G1V 0A6 Canada

**Keywords:** Criterion-referenced assessment, Validation, Family medicine

## Abstract

**Background:**

With the implementation of competency-based education in family medicine, there is a need for summative end-of-rotation assessments that are criterion-referenced rather than normative. Laval University’s family residency program therefore developed the Laval Developmental Benchmarks Scale for Family Medicine (DBS-FM), based on competency milestones. This psychometric validation study investigates its internal structure and its relation to another variable, two sources of validity evidence.

**Methods:**

We used assessment data from a cohort of residents (*n* = 1432 assessments) and the Rasch Rating Scale Model to investigate its reliability, dimensionality, rating scale functioning, targeting of items to residents’ competency levels, biases (differential item functioning), items hierarchy (adequacy of milestones ordering), and score responsiveness. Convergent validity was estimated by its correlation with the clinical rotation decision (pass, in difficulty/fail).

**Results:**

The DBS-FM can be considered as a unidimensional scale with good reliability for non-extreme scores (.83). The correlation between expected and empirical items hierarchies was of .78, *p* < .0001.Year 2 residents achieved higher scores than year 1 residents. It was associated with the clinical rotation decision.

**Conclusion:**

Advancing its validation, this study found that the DBS-FM has a sound internal structure and demonstrates convergent validity.

**Supplementary Information:**

The online version contains supplementary material available at 10.1186/s12909-021-02797-3.

## Background

Medical schools around the world are moving towards competency-based education [1], which presents assessment challenges as competencies are constructs that are difficult to operationalize [2]. Among these challenges, are the fact that competencies must be operationalized by increasing level in order to define the expected performance objectives at each stage of training [2, 3]. This need has been recognized in a number of countries, such as the United States [2] Canada [4], the United-Kingdom [5], and Australia [6]. Different terms are used to refer to these expected levels of performance such as performance levels, performance indicators, performance criteria, or benchmarks [7]. In North America, “milestones” is the commonly used term used in post-graduate medical education and it is defined as a “defined, observable marker of an individual’s ability along a developmental continuum” [8].

Milestones can be assessed at the end of each rotation [9] and it has been demonstrated that end-of-rotation assessments conducted by clinical teachers are one of the best methods for assessing the attainment of targeted competencies [10–12]. Milestones are best assessed using a criterion-referenced rather than a more traditional norm-referenced approach to assessment [13]. In the normative approach, the resident’s performance is assessed by situating it relative to that of others in the group. In contrast, in the criterion-referenced approach, performance (or level of independence) is assessed using a descriptive scale, using multiple authentic assessments situations [7]. Thus, to monitor residents’ progression, their assessment should be done using descriptive scales defining milestones, which specify the expectations at various important stages of training for several domains or contexts of practice [9]. These scales should be provided to supervisors (through residency programs) as the basis for their judgment [10].

In Canadian family medicine residency programs, there are no specific milestones defined for different levels of training, or for the end of each rotation (e.g., end of postgraduate year 1). The competency framework, the CanMEDS-FM [11] specifies the key and enabling competencies, which are what the residents are required to demonstrate at the end of their program. However, the milestones defining the expected progress at each rotation of the program have not been defined. There is therefore a need not only to develop family medicine residency milestones based on the CanMEDS-FM, but also tools to assess them at each level of training.

To address this issue, the Laval University family medicine residency program developed the Laval Developmental Benchmarks Scale for Family Medicine (DBS-FM) [12, 14] (see Additional file 1). Based on the CanMEDS-FM competency framework, the DBS-FM is an assessment tool that provides milestones and sets expectations for the development of 34 key and enabling competencies during the 26 training periods of the program. This tool focuses on a specific set of relevant competencies to be assessed at each clinical rotation.

The DBS-FM was incorporated into the family medicine residency program in 2016 as part of a gradual implementation of a competency-based curriculum that begun in the early 2010s. The introduction of this new assessment tool required training and coaching clinical teachers on how to use it. To this end, an online tutorial was offered to clinical teachers as well as on-demand coaching provided by the program director. This investment was quickly offset by the fact that clinical teachers and residents appreciate that this tool clarifies the level of competency residents are expected to attain at each training period. For this reason, results of the DBS-FM are now an essential component of residents’ progress reports.

The development and validation of the DBS-FM was informed by modern validity theory [15]. A first study insured its content validity using a Delphi methodology to identify the most salient key and enabling competencies from the CanMEDS-FM and their associated milestones [14]. A second study investigated validity evidence based on the response process upon which improvements were made to the DBS-FM [16].

The aim of this paper is to present the third validation study of the DBS-FM, which focused on the investigation of its psychometric properties. This study is important because the DBS-FM is the first milestone-based assessment tool for the CanMEDS-FM competency framework that has undergone an extensive validation process. It could therefore serve as a model for other milestone-based assessment tools in Canada and in other countries using CanMEDS as a basis for their medical competency framework [17]. In addition, we still have very little evidence about the psychometric quality of the tools developed to assess competency milestones in medical education. Studies presenting those tools, developed for other competency frameworks, provide very limited evidence on their psychometric qualities (e.g., [3, 18–20]).

## Methods

### Sample and procedures

We selected the first cohort (2016–2018) of family medicine residents assessed with the Laval DBS-FM (*n* = 106) for all the clinical rotations of their two-year program. Clinical teachers used the DBS-FM to assess their competencies at the end of each clinical rotation, totaling 1432 assessments.

### Laval developmental benchmarks scale for family medicine

The Laval DBS-FM can assess 34 enabling competencies, including 13 key (mandatory achievement) competencies, with progression milestones specified for each of them. A variable set of relevant competencies is assessed during each clinical rotation. For each of them, clinical teachers assess the level of self-directedness of residents using the following three-point scale: Supervision by direct observation / Supervision by case discussion / Independent, with specific rubrics defined for each level. Assessing the level of self-directedness can initially be challenging for clinical teachers. Indeed, our experience shows that they are used to judging residents’ performance but less so residents’ self-directedness, even if those two concepts are related. In other words, using the DBS-FM required clinical teachers to change the focus of their assessment. Depending on the competency and time period, those levels of self-directedness are considered as one of the following: early achievement, achievement at expected timing, limit for achievement of competency, or late competency achievement. In order to suggest the rotation decision (pass, in difficulty, or failure) to the evaluator, the computerized system performs a calculation based on the proportion of unachieved competencies (i.e. limit or late). This calculation takes six parameters into account: 1) a late score for one key competency or more results in a *failure*; 2) three or more late scores for non-key competencies result in a *failure*; 3) limit scores for all competencies result in an *in difficulty* decision; 4) a maximum of one late score for a non-key competency without any other late or limit results in a *pass*; 5) limit scores for all competencies, with at most one late non-key competency, lead to an *in difficulty* decision; and 6) limit scores for all key competencies only or all non-key competencies only result in a *pass*. However, the final decision as to the outcome of the rotation remains in the hands of the evaluator, who may or may not accept the system’s proposal. A competency achievement score (CAS) is also calculated, ranging from 0 to 100%, and is interpreted as the proportion of competencies for which the developmental level was assessed as “Independent” relative to the total number of competencies assessed during the clinical rotation. This score helps to keep track of residents’ progress. It is also considered in the selection process for advanced residency programs in family medicine, as a high CAS in the first year of residency is an indication of a high achievement on enabling competencies.

### Analyses

The internal structure of the DBS-FM was assessed using three sets of analyses. First, we analyzed data from the 1432 assessments with the Rasch Rating Scale Model (Andrich, 1978) in Winsteps 3.81. This model was chosen because it allows for missing data in the analysis. Therefore, it was possible to analyze the 34 items (i.e. 34 competencies) in a single model even if only item subsets were used for each clinical rotation. The Rasch analysis process was inspired by the guidelines of Tennant and Conaghan [21] and of Linacre [22]. After investigating model fit, we analyzed rating-scale functioning, dimensionality and local independence, reliability, differential-item functioning, and item targeting. Secondly, we estimated the correlation between expected and empirical item hierarchies. In fact, competencies that should be acquired early in the program according to experts consulted in a previous Delphi study [14] should be the easier items on the DBS-FM, and conversely, competencies that should be acquired late in the program according to experts should be harder items on the DBS-FM. To estimate this correlation, 31 out of the 34 competencies were used because 3 of them were modified between the Delphi study and the final version of the DBS-FM. Thirdly, to test the responsiveness of the CAS on the DBS-FM, we compared the residents’ average score for their first and second years with a paired sample t-test. Finally, we estimated the DBS-FM convergent validity with a point-biserial correlation between residents’ CAS and a dichotomous variable indicating the decision for the clinical rotation (fail /in difficulty/ pass).

## Results

### Internal structure

#### Model fit

The 34 items showed an acceptable fit to the Rasch Rating Scale Model, based on Linacre’s [22] guidelines. All items had an infit mean-square statistic between .79 and 1.49 (M = 1.03, SD = .15), and 32 had an outfit mean-square statistic between .75 and 1.43 (M = 1.07, SD = .29), with two items exceeding 1.50. Items 11 and 6 had respectively outfit mean-square values of 1.59 and 1.93. We decided nevertheless to keep both items for two reasons. First, removing them would negatively affect content validity, as these are the 34 items retained from a larger set of competencies to better represent the CanMEDS-FM framework [14]. Second, because items with infit or outfit mean-square statistics between 1.5 and 2.0 are considered “unproductive for construction of measurement, but not degrading” [22]. Infit and outfit mean-square statistics for persons had a mean of 0.97 (SD = .42) and of .98 (SD = 1.16), respectively. Out of the 1432 persons observed, 43 (3%) had a statistically significant infit or outfit value at a .01 level of significance (i.e., standardized value greater than |2.58|). They were removed from subsequent analyses. Upon removal, mean item and person fit statistics improved slightly. Items infit and outfit mean-square values were thereafter respectively 1.01 (SD = 0.12) and 1.00 (SD = 0.38), while person infit and outfit mean-square values were respectively 0.98 (SD = 0.36) and 0.90 (SD = 0.89).

#### Rating scale functioning

**Fig. 1:**
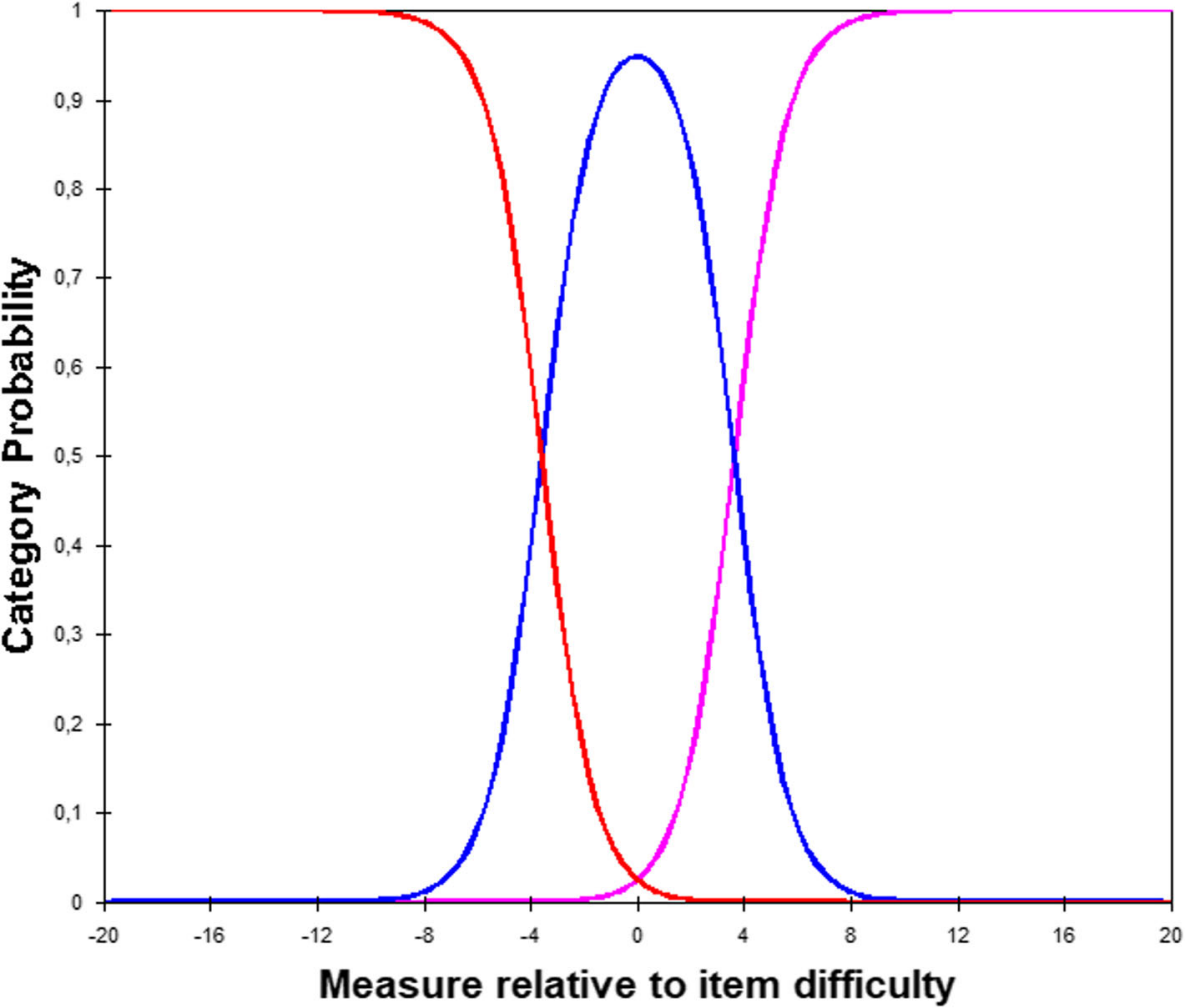
Option characteristic curves for the 3-point scale

Option characteristic curves are illustrated in Fig. 1. Analysis of the rating scale structure was carried out using Linacre’s [23] eight guidelines, summarized in Table 1. Guidelines 1, 3, 4, 5, 7 were respected, while guidelines 2, 6, and 8 were not. Non-respect of the second guideline (Regular observation distribution) reflected the fact that only 0.2% of the observations received the lowest rating (1 = Supervision by direct supervision), while the majority (85.7%) of the observations received the highest rating (3 = Independent). Regarding the sixth guideline (Ratings imply measures, and measures imply ratings), the low congruence between ratings and measures concerned the lowest rating (option 1) and therefore relied on only 54 observations for this estimate. Non-respect of the eighth guideline (Step difficulties advance by less than 5.0 logits) implies large steps on the latent variable between rating options and therefore less measurement precision.

**Table 1:** Analysis of the rating scale structure using Linacre’s [23] eight guidelines

Linacre’s (2004) guidelines	Result
1. At least 10 observations of each category	There were at least 10 observations per response option (54 observations in the first option; 3615 in the second; and 22,023 in the third).
2. Regular observation distribution	Distribution of observations across response options was irregular, meaning that option 3 was clearly the most frequent option, followed by option 2, while option 1 was seldom chosen.
3. Average measures advance monotonically with category	Average ability estimates advanced monotonically with options going from −1.10 logits (option 1) to 2.77 logits (option 2) and then to 6.59 logits (option 3).
4. Outfit mean-squares less than 2.0	Infit and outfit indices were acceptable, all comprised between .99 and 1.30.
5. Step calibrations advance	Step calibrations advanced, indicating no disordered thresholds. The step between option 1 and 2 was estimated at −3.61 logits, and the step between option 2 and 3 was estimated at 3.61 logits.
6. Ratings imply measures, and measures imply ratings	Congruence between measures and ratings as well as between ratings and measures was generally good. It varied between 66 and 93% for options 2 and 3. For option 1, the congruence between measures and ratings was acceptable at 55%, but the congruence between ratings and measures was at 11%.
7. Step difficulties advance by at least 1.4 logit	The distance of 7.22 logits between the two steps was larger than 1.4 logits.
8. Step difficulties advance by less than 5.0 logits	The distance of 7.22 logits between the two steps was larger than 5 logits.

#### Dimensionality and local Independence

A principal residuals component analysis showed that the first dimensions had an Eigenvalue of 33.3 and explained 49.5% of score variability. The second dimension had an Eigenvalue of 1.9 and explained 2.8% of score variability. The second dimension having a strength of less than two items, the structure of the DBS-FM was considered unidimensional. Regarding local independence, the largest standardized residual correlation between the items had a value of .48 (between items 1 and 2), indicating that the maximum amount of shared variance between two items was 23%. Items were therefore considered locally independent.

#### Differential item functioning

We tested the invariance of the measurement scale between year 1 and year 2 observations. This was done by investigating for the presence of differential item functioning (DIF) based on residency level (year 1 versus year 2) using Welch’s t-test. A Bonferroni correction was applied to guard against the inflation of type 1 error because this analysis resulted in 34 tests, i.e. one for each item. The alpha level of statistical significance was therefore set at .05/34 = .001. Two items (21 and 22) showed significant DIF, both being easier for year 2 residents. The Item 21 (Clinical expertise – Technical gestures) parameter estimate was 3.05 logits for year 1 residents and 1.84 logits for year 2 residents, with an estimated difference of 1.22 logits between the two. The Item 22 (Clinical expertise – Investigation and treatment) parameter estimate was 2.38 logits for year 1 residents and 1.39 logits for year 2 residents, with an estimated difference of .98 logits between the two. To test the impact of these DIF on ability estimates, we correlated resident ability estimated with and without these two items. The correlation between these two score sets was 0.99.

#### Reliability of CASs

**Fig. 2:**
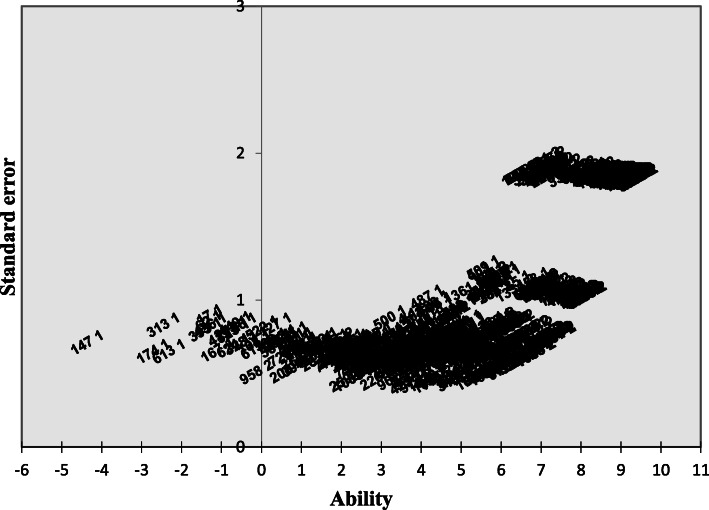
Standard error of measurement relative to estimated Rasch ability level of residents

The reliability of residents’ CASs was estimated at .83 for observations not having an extreme score (*n* = 752) (i.e. ability parameter of 7.00 logits or lower), and at .66 (*n* = 1389) when including an analysis of the 637 residents having an extreme score. As can be seen in Fig. 2 below, the extreme scores, especially those at the top of the scale, have the highest standard error or, in other words, the lowest measurement precision. Classical reliability estimates for the subsets of items used in the different clinical rotations, using Cronbach’s alpha, were between .76 and .93.

#### Item targeting

**Fig. 3:**
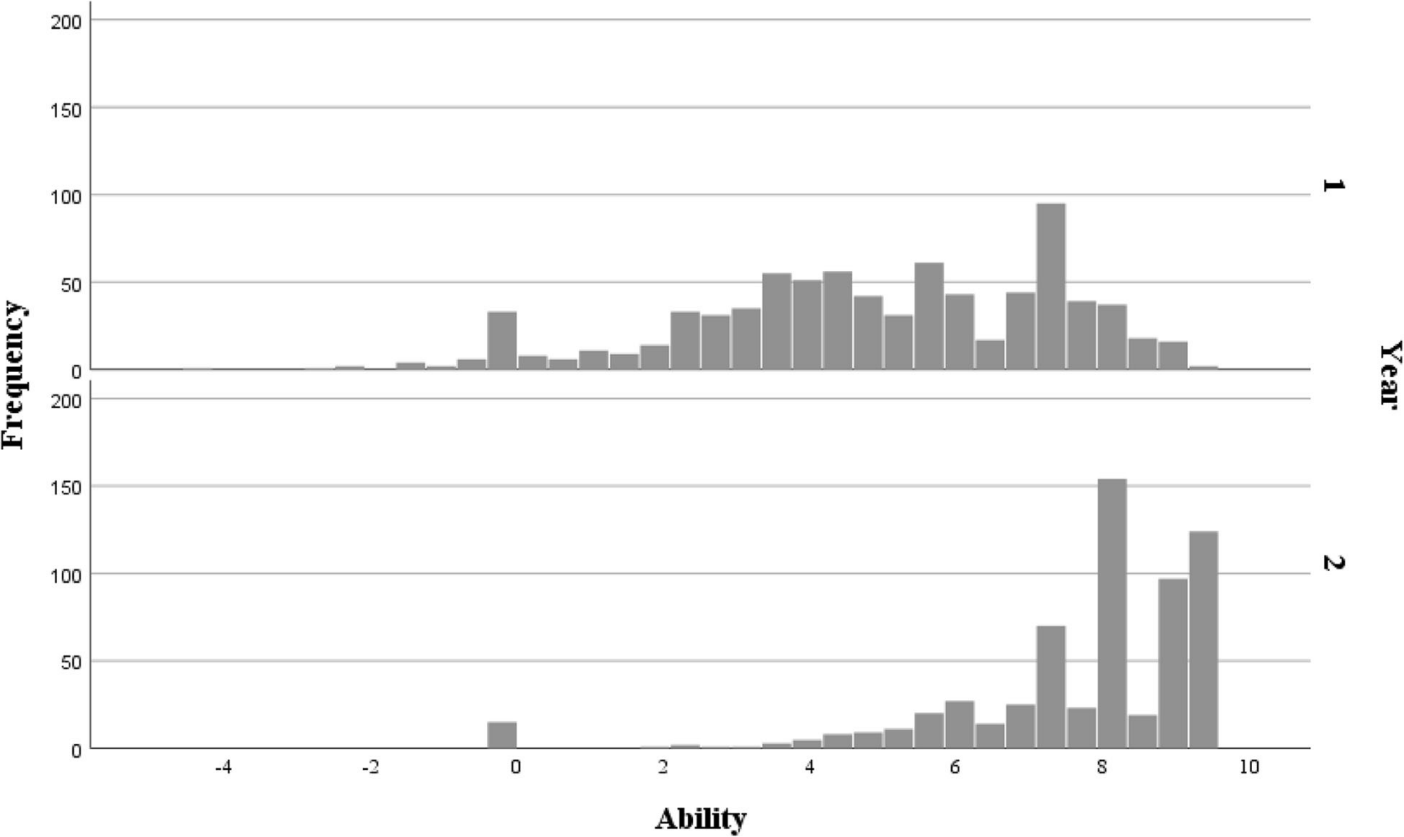
Distribution of the Rasch ability parameters for year 1 (top) and year 2 (bottom) residents

**Fig. 4:**
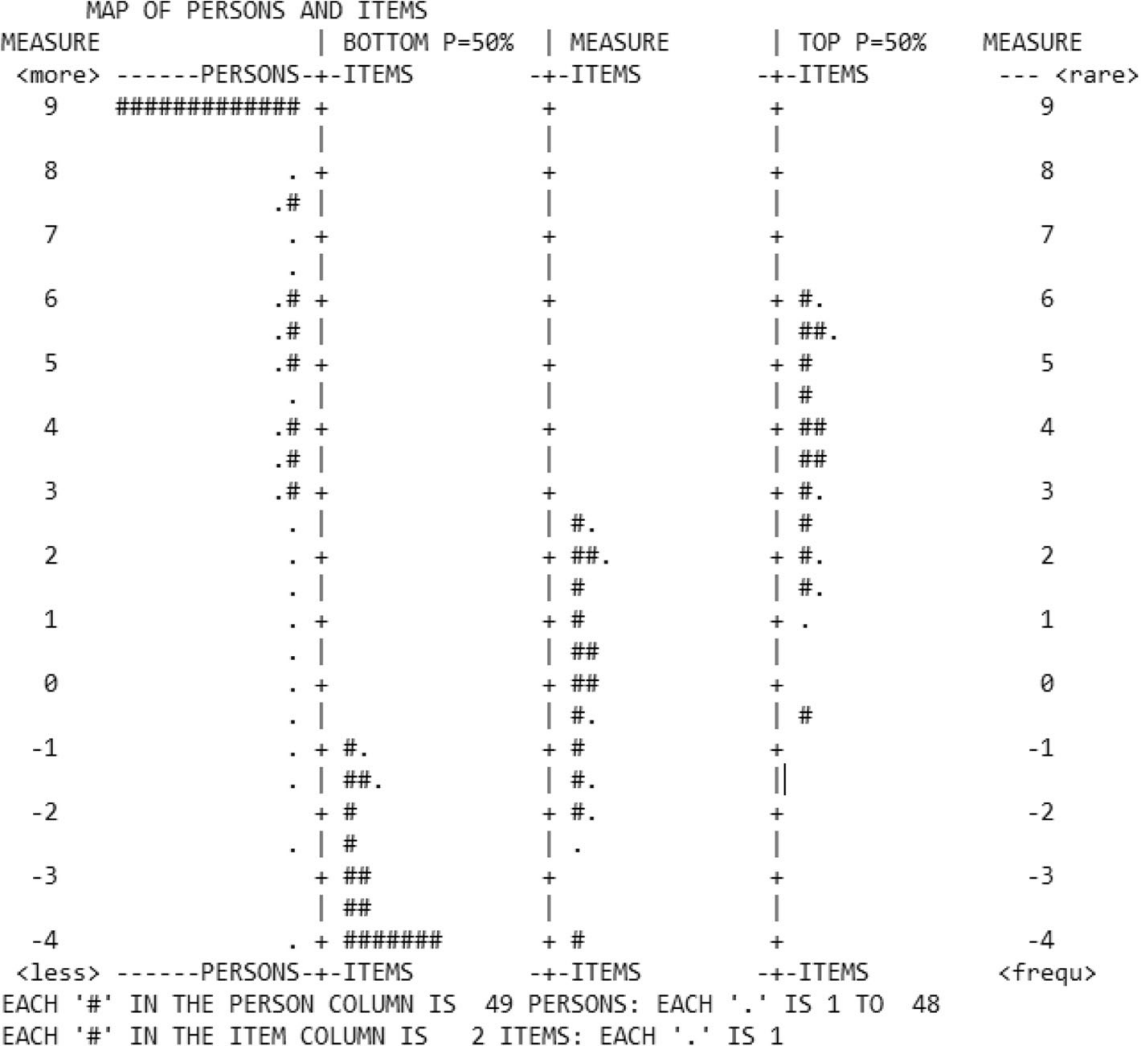
Wright map of persons and items parameters

Residents’ ability parameters ranged from − 4.33 to 9.45 logits (M = 6.34 logits, SD = 2.43). More precisely, as illustrated in Fig. 3, ability parameters for year 1 residents ranged from − 4.33 to 9.45 logits (M = 4.89 logits, SD = 2.46) (*n* = 803 assessments), and from − 0.09 to 9.45 logits (M = 7.75 logits, SD = 1.85) for year 2 residents (*n* = 629 assessments). In comparison, difficulty parameters for the 34 items of the DBS-FM ranged from − 4.24 to 2.72 logits (M = 0.00 logits, SD = 1.79). The Wright map (Fig. 4) shows the location of the candidates (“person” column) and items (“measure” column) relative to each other on the latent variable. The “BOTTOM *P* = 50%” column shows the Rasch-Thurstone thresholds for the lowest rating (option 1) on each item, where the probably of being rated as “1″ or higher is 50%. The “TOP P = 50%” column shows the Rasch-Thurstone thresholds for the highest rating (option 3) on each item, where the probably of being rated 3 or below is 50%. The distance between the bottom and upper Rasch-Thurstone thresholds is the operational range of the scale, in other words the latent variable range where the scale is able to discriminate between different competency levels, i.e. between approximately − 8.00 and 7.00 logits. Therefore, the scale cannot discriminate between the highest scoring residents, located between 7.00 and 9.45 logits. For year 1 residents, 232 (32%) out of the 803 assessments were higher than 7.00 logits. For year 2 residents, 489 (68%) of the 629 assessments were higher than 7.00 logits.

#### Item hierarchy

The expected item hierarchy corresponded to the ordering of competencies by time of expected achievement by the 28 experts at the last phase of the Delphi study [14]. This ordering was highly reliable, both the Generalizability coefficient [24] and the Dependability index [25] being .91. The empirical item hierarchy estimate was also reliable (Rasch item reliability = 0.99). The correlation between the expected item hierarchy according to experts and the empirical item hierarchy estimated by the Rasch item difficulty parameters was.78, *p* < .0001.

#### Global score responsiveness

**Fig. 5:**
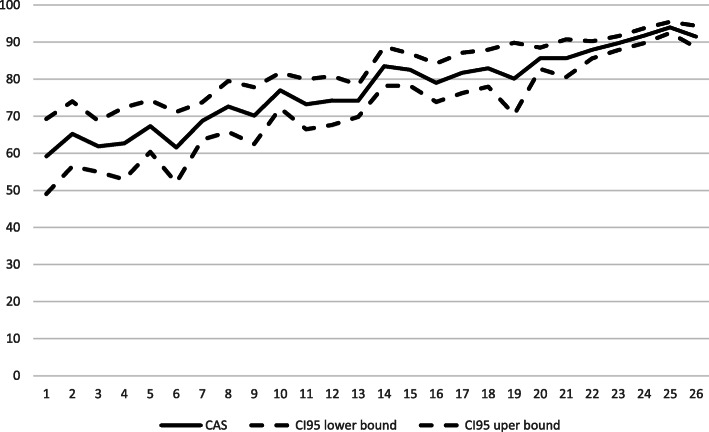
Average CAS with 95% confidence intervals for the 26 periods of the residency program

Figure 5 shows the average CAS on the DBS-FM with 95% confidence intervals for the 26 periods of the residency program. The average CAS was .71 (SD = .18) for year 1 residents (clinical rotations 1 to 13) and .83 (SD = .10) for year 2 residents (clinical rotations 14 to 26). A paired sample t-test showed that the difference between the average CAS for year 2 and year 1 residents is statistically significant, *t*(94) = − 7.52, *p* < .0001. Using the Rasch ability parameters rather than the CASs yielded similar results, *t* (1427.6) = − 25.00, *p* < .0001.

**Fig. 6:**
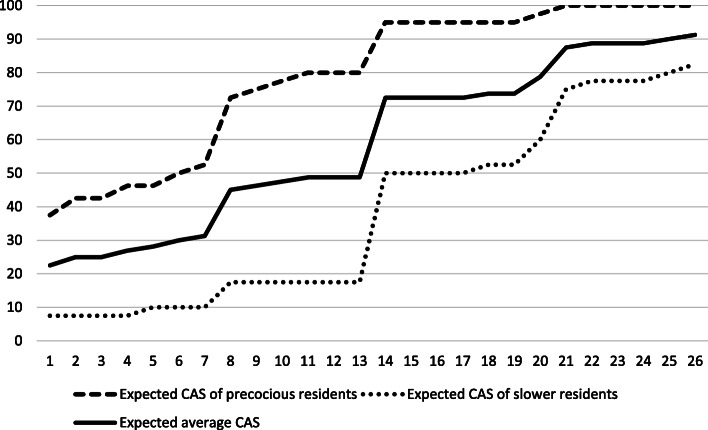
Expected CAS

However, the difference between those 2 years is lower than expected. The expected CAS (Fig. 6) for the first year of residency varied between .23 and .49 for an average student, which is much lower than the observed CAS, which varied between .59 and .74. The expected CAS for year 2 residents varied between .73 and .91, which is comparable to the observed CAS that varied from .74 to .94.

### Convergent validity

Results from the point-biserial correlation, *r* = −.28, *p* < .0001, show that the CAS was significantly associated with being classified as “pass” or “in difficulty or failure.” In other words, having a low CAS was associated with a higher probability of an “in difficulty or failure” decision for a clinical rotation.

## Discussion

The DBS-FM is a criterion-referenced, milestone-based, assessment tool based on the CanMEDS-FM used to assess family medicine residents at the end of each clinical rotation. In this validation study, we used modern and classical psychometric analyses to gather empirical evidence on its internal structure and relation to another variable. To the best of our knowledge, it is the first study to extensively explore the psychometric qualities of a milestone-based tool designed for the assessment of residents. Indeed, previous studies focused essentially on content validity, variability of scores and their capacity to show residents’ progression (e.g., 3, 18, 19, 20). Results of the study show that milestone-based assessments of residents can be reliable and discriminate between competency levels and stages of residency, and can be summarized into a global latent competency score. In addition, the DBS-FM can be used by other medical schools as a model or an example of a milestone-based assessment tool that has undergone extensive validation. It could therefore contribute to filling an identified gap in the adoption and implementation of competency milestones in residency programs [2].

Analyses of the internal structure showed that the DBS-FM can be considered as unidimensional with no locally dependent items. Consequently, it is appropriate to summarize residents’ competency level using a single synthetic score, and this score is sensitive enough to reflect residents’ progression between their first and second year, while individual items can be used to provide more directed feedback. The ability to summarize competency levels using a single score on a latent construct is also compatible with the conceptual view that a competence is a general quality or attribute that is not directly observable [26]. In addition, the empirical item hierarchy supports the adequacy of the ordering of milestones by experts consulted in a previous study [14]. The correlation of 0.78 indicates that the expected and empirical item hierarchies share 61% of variance. We consider this to be relatively high, as experts usually struggle to guess the difficulty level of items [27].

Internal structure analyses also showed that the Classical Test Theory reliability of the subset of items used for the different clinical rotations varies between acceptable (α = .73) and very good (α = .93) [28]. The reliability of the 34 items of the DBS-FM, estimated by the Rasch model, is good (.83) for non-extreme scores (i.e. scores lower than 7.00 logits). However, reliability drops (.66) with the inclusion of extreme scores due to their large degree of measurement error. This means that the DBS-FM cannot reliably discriminate between the highest observed competency levels (i.e., 7.00 logits and higher), resulting in a large standard error of measurement for the highest scores. In other words, the item targeting is adequate for the goal of measuring low and intermediate competency levels, but not for measuring the highest levels. This is in line with the aim of the DBS-FM, which is not to discriminate among solid levels of competency, but to help the program ensure that every resident achieves the minimal competency level needed for independent professional practice and to identify those who do not meet this minimal level. It should also be mentioned that criterion-referenced assessments have long been known for having lower item variances than normative-referenced assessments because scores are more concentrated at the higher end of the notation scale [29–31].

If one needed to reliably discriminate between the highest competency levels, some solutions could be envisioned. For example, harder items (i.e. competencies achieved at the end of the two-year program or competencies achieved at the end of the two-year program only by some, but generally achieved later by most) could be added to the DBS-FM. In addition, the highest rating (option 3) on the rating scale could be split into two or three options, with the highest option going beyond “Independent.” The large distance in logits between the two steps of the rating scale suggests that there is space on the latent competency variable for a finer-grained rating scale. For instance, a rating scale similar to the O-SCORE could be considered [32]. The O-SCORE has 5 levels that reflect a finer grained progression toward complete independence. Such strategies would also help to identify top performers for promotion or selection purposes.

We also observed differential item functioning for items 21 (Clinical expertise – Technical gestures) and 22 (Clinical expertise – Investigation and treatment) when comparing year 1and year 2 residents. Both items relate to clinical expertise and were harder for year 1 students than for year 2 students when the ability level remained constant. Our hypothesis is that at a similar ability level, year 1 residents are still not as good as year 2 residents when it comes to investigation and treatment as well as to technical gestures. The two differential item functionings did not have a practical impact because the correlation between the residents’ ability parameters estimated with and without these items was 0.99. Therefore, differential item functioning does not pose a threat to the validity of the interpretation of residents’ scores.

The CAS showed sensitivity to change and made it possible to detect a statistically significant difference between the performance of year 1 and year 2 residents. This result is consistent with that of other studies that also found that milestone-based assessments of residents can reflect residents’ growth over time or distinguish between stages of training [20, 33]. However, in the present study, the difference between year 1 and year 2 residents was lower than predicted. The prediction was that year 1 residents would have much lower CAS (between .23 and .49, rather than the observed .59 to .74) and would show a relatively big increase of .24 (from .49 to .73) in their CAS between the 13th and 14th period, representing the transition between year 1 and year 2, similar to what was observed by Goldman et al. [20]. The empirical data show that year 1 residents have better CAS than expected and that the transition from year 1 to year 2 is much more gradual. This gradual increase in competency level throughout residency training was also observed in another study [32]. A possible explanation for these divergent results in the literature is that the progression of residents’ competency levels could be highly dependent on the program.

The DBS-FM has convergent validity when correlated with the clinical rotation decision (“pass” vs “fail/in difficulty”). A higher CAS was associated with a higher probability of being classified as “pass,” while a lower CAS was associated with a higher probability of being classified as “fail” or “in difficulty.” Stated differently, the CAS demonstrates decision consistency with pass/fail decisions. This is a necessary quality to ensure the credibility of the assessment.

There are some limits to this study. First, although it seems plausible that these results should be similar for the next cohorts of family medicine residents, they cannot be automatically generalized. Variations between cohorts, between assessors, or interaction effects between cohorts and assessors, for example, could lead to some variations in its psychometric properties. It will therefore be necessary to monitor the psychometric properties of the DBS-FM for future cohorts. Second, the DBS-FM can be used as a model by other family medicine programs, but it will need to be adapted to the reality of those programs to ensure its validity. Third, differential item functioning was tested for year of residency, but not for gender, due to the anonymous nature of the data. However, the milestones for the acquisition of some competencies could differ between males and females, which would result in differential item functioning. Fourth, when investigating the DBS-FM’s relations to other variables, we tested its convergent validity, but not its criterion-related validity. We originally planned to test its predictive validity by comparing the mean CAS on the DBS-FM for residents who passed and those who failed the Certification Examination in Family Medicine of the College of Family Physicians of Canada. But the number of residents who failed this certification exam was too low to run a statistical analysis.

## Conclusions

The DBS-FM has a sound internal structure and good convergent validity. It is the first criterion-referenced assessment tool based on the CanMEDS-FM competency framework that is used to assess milestones and that has undergone an extensive validation process. It could therefore serve as a model for other milestone-based assessment tools. Future studies are needed to investigate the validity of criterion-referenced milestone-based assessment tools in the context of formative assessment.

## Supplementary Information

**Additional file 1.**

## Data Availability

The datasets generated during and/or analyzed during the current study are not publicly available due to the fact that they contain students’ assessment data that the corresponding author is not authorized to share.

## Abbreviations

DBS-FM: Developmental Benchmarks Scale for Family Medicine
CanMEDS-FM: The competency framework for Canadian family physicians
CAS: Competency achievement score
M: Mean
SD: Standard deviation
Infit: Inlier-pattern-sensitive fit statistic
Outfit: Outlier-sensitive fit statistic
Logit: Log-odds unit
DIF: Differential item functioning
r: Point-biserial correlation coefficient
