# Supplementary material for: Psychometric validation of the Laval developmental benchmarks scale for family medicine
Source: BMC Med Educ. 2021 Jun 27;21:357. doi: 10.1186/s12909-021-02797-3 (PMC8237442; doi:10.1186/s12909-021-02797-3)
Supplement: Supplementary file 1 — Additional file 1. [file 12909_2021_2797_MOESM1_ESM.pdf]

Developmental Benchmarks Scale for Family Medicine

Expected timeline to achieve “independent” entrustment level during residency training

UNIVERSITÉ LAVAL

Faculté de médecine
Département de médecine familiale
et de médecine d'urgence

Developmental Benchmarks Scale for Family Medicine

Expected timeline to achieve "independent" entrustment level during residency training

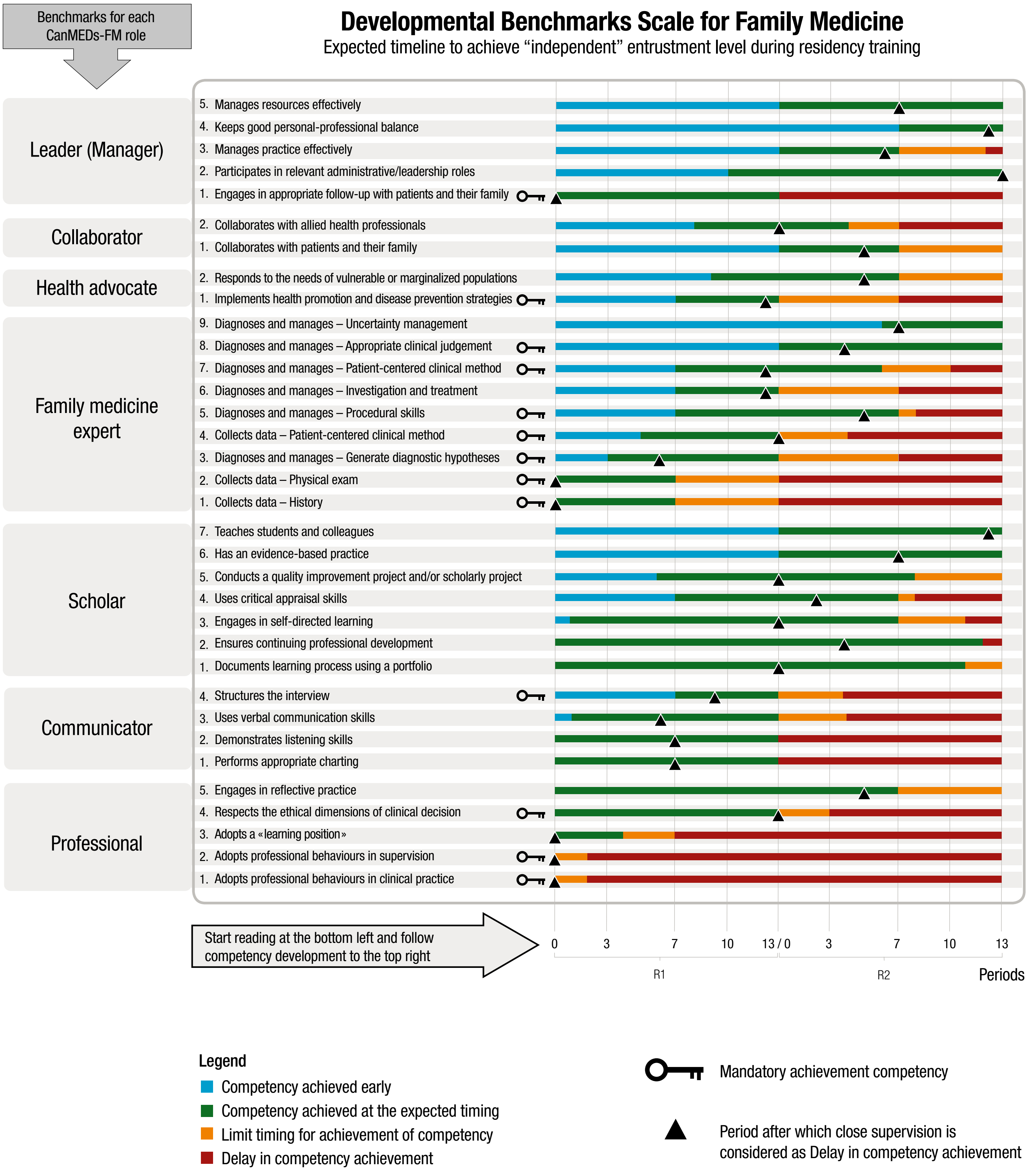

		Close supervision	Distant supervision	Independent
Professional	1. Adopts professional behaviours in clinical practice	In clinical practice, demonstrates behaviours that do not meet the requirements of honesty, compassion, respect for patients, selflessness, commitment to patient wellness, or appropriate interpersonal professional relationship boundaries.	In clinical practice, needs coaching to adopt behaviors that meet the requirements of honesty, compassion, respect for patients, selflessness, commitment to patient wellness, or appropriate interpersonal professional relationship boundaries.	In clinical practice, spontaneously adopts behaviors that meet the requirements of honesty, compassion, respect for patients, selflessness, commitment to patient wellness, or appropriate interpersonal professional relationship boundaries.
	2. Adopts professional behaviours in supervision	During supervision, adopts behaviors that do not meet the requirements of honesty, reliability, respect and appropriate professional relationship boundaries.	In clinical supervision, needs coaching to adopt behaviors that meet the requirements of honesty, reliability, respect and appropriate professional relationship boundaries.	In clinical supervision, spontaneously adopts behaviors that meet the requirements of honesty, reliability, respect and appropriate professional relationship boundaries.
	3. Adopts a « learning position » in which assessment is perceived primarily as formative/ integrated into the normal physician training stream	Hides or covers up areas for improvement, chooses the easy/mastered clinical situations and avoids difficult situations. Displays a closed and defensive attitude, tends to justify him/herself when facing criticism, denies difficulties, and minimizes negative feedback.	With appropriate coaching, acknowledges own limits and asks for help if needed. Welcomes criticism which is immediately perceived as constructive, questions the unfavorable comments in order to better understand their scope.	Acknowledges own limits and asks for help if needed. Spontaneously addresses encountered difficulties, reflects a desire to progress and an openness in taking appropriate risks to get there.
	4. Respects the ethical dimensions of clinical decision	Explains little or does not explain the benefits and risks of proposed interventions and the consequences of not intervening; reveals personal information against the expressed will of the patient or speaks of patients in public environment; does not respect patient decisions and autonomy.	Stereotypically explains the benefits and risks of proposed interventions and the consequences of not intervening; respects patient privacy, respects patient autonomy in their decision making.	Explains in a manner appropriate to the patient the benefits and risks of proposed interventions and the consequences of not intervening to allow a free and informed decision; respects the privacy of patients; respects and promotes patient autonomy in their decision making.
	5. Engages in reflective practice	Does not recognize the factors that could have an impact on consultations. Does not take time to reflect on events and actions in clinical practice	Recognizes the factors that could have an impact on consultations, but does not consider the implications for patient or self. With coaching, reflects on the events occurring in his/her practice, especially critical incidents, to refine knowledge of him/herself.	Recognizes the factors that could have an impact on consultations and works to resolve them before meeting with patients. Able to spontaneously reflect on the events occurring in his/her practice, especially critical incidents, to refine knowledge of him/herself.
Communicator	1. Performs appropriate charting	Maintains unclear, inaccurate records, incompletely reflecting consultation, or inconsistent with professional regulations. Does not complete records contemporaneously.	With appropriate coaching, maintains clear and accurate records, consistent with professional regulations. With few exceptions, completes records within 24 hours of patient appointment.	Spontaneously maintains clear and accurate records, consistent with professional regulations. With few exceptions, completes records within 24 hours of patient appointment.
	2. Demonstrates listening skills	Inattentive or distracted when taking a patient history; misses nonverbal cues provided by the patient.	Listens properly to patient answers and grasps nonverbal cues, without adjusting data collection and analysis accordingly.	Actively listens to patient answers and grasps nonverbal cues to adjust data collection and analysis accordingly.
	3. Uses verbal communication skills	Seems to be misunderstood by the patient; often holds a conversation not well adapted to the patient; pays little attention to interview techniques. Has an intuitive approach to induce a change in behavior, break bad news or manage a difficult patient.	Sometimes, seems misunderstood by the patient; occasionally holds a conversation not well adapted to the patient; demonstrates appropriate use of some interview techniques. When prompted, uses accepted strategies / communication models to induce a change in behavior, break bad news or manage a difficult patient.	Seems well understood by the patient; holds a conversation well adapted to the patient; demonstrates appropriate use of some interview techniques. Spontaneously uses accepted strategies / communication models to induce a change in behavior, break bad news or manage a difficult patient.
	4. Structures the interview	Does not list patient chief complaints, or ignores them; does not contract with patient at the beginning of the interview, struggles to control the interview, or rigidly controls the interview.	With appropriate coaching, explores patient chief complaints early in the interview; when prompted, balances his agenda with that of the patient; needs regular guidance to control the interview effectively.	Explores all patient chief complaints early in the interview and spontaneously balances his agenda with that of the patient; controls the interview with appropriate fluency.
Scholar	1. Documents learning process using a portfolio	Superficially documents (or does not document) learning process through the development of a portfolio, with few features relevant to his/her level of training.	Partially documents learning process through the development of a portfolio, displaying several features relevant to his/her level of training.	Specifically documents learning process through the development of a portfolio, displaying all features relevant to his/her level of training.
	2. Ensures continuing professional development	Is often missing academic sessions, has not completed the mandatory self-learning modules expected at his/her level of training, does not participate in the expected simulation workshops. Does not attend continuing professional development activities.	Participates in the academic sessions, has completed some of the mandatory self-learning modules expected at his/her level of training, participated in the expected simulation workshops. Explores some continuing professional development activities (ex.: conferences).	Participates actively and assiduously in the academic sessions, has completed all the mandatory self-learning modules expected at his/her level of training, participated in the expected simulation workshops. Attends continuing professional development activities thoughtfully chosen to consolidate clinical competence.
	3. Engages in self-directed learning	Mostly refers to preceptor's opinion to find answers to clinical questions.	Reads reviews or expert opinion articles to answer clinical questions.	Consults relevant scientific literature (practice guidelines, original research, etc.) to find detailed answer to clinical questions.

		Close supervision	Distant supervision	Independent
Scholar (continued)	4. Uses critical appraisal skills	Critically appraises literature data with difficulty.	Critically appraises literature, seeks assistance in the interpretation of some of scientific aspects of the article.	Critically appraises literature, with appropriate interpretation of the scientific aspects of the article.
	5. Conducts a quality improvement project and/or scholarly project	Unable to identify the project objective, conducts a superficial literature search in online databases, ignores which methodology to use or is not able to discuss findings appropriately.	Needs close coaching to identify the project objective, to conduct a literature search in online databases, to define methodology and to discuss findings appropriately.	Identifies the project objective, conducts literature searches in online databases, defines methodology and discuss findings appropriately, requesting help only for specialized aspects of the project.
	6. Has an evidence-based practice	Rarely considers available evidence in the use of diagnostic and therapeutic tools.	With appropriate coaching, considers available evidence in the use of diagnostic and therapeutic tools. Tends to directly apply conclusions from critical appraisal without ensuring applicability to the patient on an individual basis.	Spontaneously considers available evidence in the use of diagnostic and therapeutic tools. Adjusts conclusions from critical appraisal to ensure applicability to the patient on an individual basis.
	7. Teaches students and colleagues	Teaches or supervises learners intuitively based on his/her past learning experience, without adjusting to students' needs.	Incorporates some teaching strategies in small group teaching or clinical supervision in order to vary approaches, occasionally adjusting to students' needs.	Uses varied teaching strategies in small group teaching or clinical supervision to encourage active learning most often adjusted with students' needs.
Family medicine expert	1. Collects data – History	Assesses patients in an exhaustive but stereotyped way, may seem disorganized or unfocused, or to incompletely assess the problems. Misses key features, does not adjust to cues arising during the interview»	Performs more or less focused patient assessment, defines problem well, but spends excessive time assessing less relevant information.	Selectively adjusts patient assessment by focusing on relevant information.
	2. Collects data – Physical exam	Performs a physical examination in a thorough but stereotypical fashion; is unfocused, or sometimes not reproducible, may use incorrect or inappropriate technique. Does not interact with the patients during the examination.	Performs a thorough but relatively focused and reproducible physical examination. Common examination techniques performed correctly. Interacts with the patient during the examination.	Performs a focused and reliable physical examination, including specialized examination techniques when relevant. Comfortably interacts with the patient during the examination.
	3. Diagnoses and manages – Generate diagnostic hypotheses	Does not identify red flags or serious conditions. Does not look for key features (including relevant negatives); is cursory in formulating and justifying hypotheses that guide patient assessment; generates an incorrect differential or focuses too quickly on a single hypothesis (premature closure).	For common primary care complaints, identifies most key features for generating a realistic differential diagnosis (sometimes too broad or incomplete).	For common primary care complaints, considers diagnostic hypotheses based on prevalence and risk, but quickly focuses a few specific differential diagnoses according to identified key features. Can be less familiar with atypical presentations.
	4. Collects data – Patient-centered clinical method	Rarely explores patient experience and context during consultations.	Explores patient experience and context in most consultations, but usually in a stereotyped manner.	Adapts exploration of patient experience and context to clinical situation; clearly and empathetically reflects the challenges of experience and context to the patient.
	5. Diagnoses and manages – Procedural skills	Selects inappropriate intervention, does not obtain consent or obtains incomplete consent, improperly prepares for intervention, applies incorrect technical skills; unsafely discards hazardous materials; plans inappropriate follow-up.	Selects appropriate intervention, obtains consent based on correct information; prepares properly with appropriate coaching; demonstrates correct technical skills with appropriate coaching; safely discards hazardous materials; plans for appropriate follow-up.	Selects appropriate patient-centered intervention, obtains patient-centered consent; prepares properly; spontaneously demonstrates correct technical skills while paying attention to patient comfort; safely discards hazardous materials; tailors follow-up to patient needs.
	6. Diagnoses and manages – Investigation and treatment	Chooses inappropriate or generic/stereotyped investigations and treatments rather than tailoring them to patient's situation.	For common primary care complaints, identifies, with appropriate coaching, investigations and treatments tailored to the patient's situation.	For common primary care complaints, spontaneously identifies investigations and treatments tailored to the patient's situation.
	7. Diagnoses and manages – Patient-centered clinical method	Adopts a rather unilateral and paternalistic discourse. Is rarely inclined to give patients and their families information about the problem and associated management.	Must be coached to encourage discussion, questions and feedback from patient. Teaches patients and their families generic information regarding the patient problem and associated management. When reminded, involves the patient to find common ground about management (shared decision making).	Spontaneously encourages discussion, questions and feedback from patient. Sensitively adapts teaching to patients and their families. Usually involves patient spontaneously to find common ground about management (shared decision making).
	8. Diagnoses and manages – Appropriate clinical judgement	Makes clinical decisions where the proposed diagnosis and management are inconsistent with the symptoms and signs of the patient. Does not prioritize assessment or management in light of the urgency of a clinical situation.	With appropriate coaching, makes logical decisions linking the identified clinical signs and symptoms, the diagnosis and the proposed management. With supervisor's help, prioritizes work-up or management in light of the urgency of a clinical situation.	Makes logical decisions linking the identified clinical signs and symptoms, the diagnosis and the proposed management. Spontaneously prioritizes work-up or management in light of the urgency of a clinical situation.
	9. Diagnoses and manages – Uncertainty management	Shows a marked insecurity when facing uncertainty, or does not recognize a situation in which he/she should feel uncertain.	Manages clinical problems in a context of uncertainty mainly by consulting preceptors.	Manages clinical problems in a context of uncertainty not only by consulting preceptors, but also consulting other appropriate sources of information such as colleagues and by encouraging shared decision making with the patient.

		Close supervision	Distant supervision	Independent
Health advocate	1. Implements health promotion and disease prevention strategies	Rarely discusses health promotion and disease prevention strategies with patients, or suggests inappropriate strategies not tailored to patient's need.	When prompted, discusses health promotion and disease prevention strategies with patients.	Spontaneously implements health promotion and disease prevention strategies suitably adapted to patient needs.
	2. Responds to the needs of vulnerable or marginalized populations	Rarely identifies or, declines to take care of some vulnerable (elderly, mental health, chronic illness, etc.) or marginalized (immigrant, homeless, indigenous, etc.) populations.	With appropriate coaching, responds appropriately to the needs of vulnerable (elderly, mental health, chronic illness, etc.) or marginalized (immigrant, homeless, indigenous, etc.) populations.	Responds appropriately to the needs of vulnerable (elderly, mental health, chronic illness, etc.) or marginalized (immigrant, homeless, indigenous, etc.) populations.
Collaborator	1. Collaborates with patients and their family	Rarely consults with patients, family members and / or caregivers (when relevant) to guide interventions.	When prompted, consults with patients, family members and / or caregivers (when relevant) to guide interventions.	Spontaneously consults with patients, family members and / or caregivers (when relevant) to guide interventions.
	2. Collaborates with allied health professionals	Over-seeks or under-seeks consultation with other health professionals. Writes inappropriate consultation requests with a nonspecific question, insufficient or non-targeted clinical information, or uses confusing language.	Wisely refers patients for consultation with other health professionals without considering the patients preferences or specific circumstances, or using personal knowledge of the referral network. Writes appropriate consultation requests with a clear question, relevant and targeted clinical information and appropriate language.	Wisely adjust patient referrals considering the patients preferences or specific circumstances, or using personal knowledge of the referral network. Writes thoughtful consultation requests incorporating all relevant medical and psychological elements and plans the expected work-up before consultation.
Leader (manager)	1. Engages in appropriate follow-up with patients and their family	Does not ensure follow-up of investigations or does not plan reasonable follow-up with patients and their families.	With appropriate coaching, ensures follow-up of investigations and plans reasonable follow-up with patients and their families.	Spontaneously ensures follow-up of investigations and is proactive to plan quick follow-up with patients and their families if needed.
	2. Participates in relevant administrative/ leadership roles	Requires close monitoring to carry out administrative roles (communications when out of office, team work, activity planning) relevant to his/her clinical practice. Needs reminding to participate in working group meetings.	When prompted, carries out administrative roles (communications when out of the office, team work, activity planning, etc.) relevant to his/her his clinical practice. Actively participates in working group meetings.	Spontaneously carries out administrative roles (communications when out of the office, team work, activity planning, etc.) relevant to his/her his clinical practice. Able to lead working group meetings.
	3. Manages practice effectively	Inconsistently or incompletely prepares before patient encounters. Needs to be guided closely to use information technologies to provide patient care. Evaluates patients without thinking of workflow.	With appropriate coaching, adequately prepares before patient encounters. Uses information technologies more or less efficiently to provide patient care. Takes about twice as long as a physician new to independent practice.	Spontaneously prepares adequately before patient encounters. Uses information technologies efficiently to provide patient care. Evaluates patients with a rate consistent with early practice.
	4. Keeps good personal-professional balance	Presents difficulty in prioritizing various professional obligations when facing multiple requirements. Takes too much or not enough time to meet his/her personal needs. When a conflict between professional and personal activities is brought to his/her attention, does not take it into account nor adjust accordingly.	With appropriate coaching, prioritizes various professional obligations when facing multiple requirements. Usually protects a suitable time to meet his/her personal needs. When a conflict between professional and personal activities is brought to his/her attention, discusses it on demand and adjusts accordingly.	Spontaneously prioritizes various professional obligations when facing multiple requirements. Protects a suitable time to meet his/her personal needs. When a conflict between professional and personal activities is brought to his/her attention, spontaneously discusses it and adjusts accordingly.
	5. Manages resources effectively	Minimally considers the consequences of his/her investigations / management decisions and associated costs for the health system.	With appropriate coaching, generally considers the consequences of his/her work-up / management decisions and associated costs for the health system.	Spontaneously considers the consequences of his/her work-up / management decisions and associated costs for the health system.

Designed by the family medicine residency program, Université Laval with the financial support of Fonds Gilles-Cormier, Université Laval.

Miriam Lacasse MD MSc CCFP, Christian Rheault MD CCFP, Isabelle Tremblay MSc, Jean-Sébastien Renaud PhD, Frédéric Coché MEd, Annie St-Pierre MD PhD CCFP, Johanne Théorêt MD MA CCFP, Sylvie Tessier MD CCFP, Louise Arsenault MA, Marie-Lee Simard, Caroline Simard PhD(c), Isabelle Savard PhD, Josette Castel MD MSc CCFP MF, Luc Côté PhD.
Département de médecine familiale et de médecine d'urgence, Université Laval, Québec (Canada).

For information, please contact :

Miriam Lacasse, MD MSc CCFP
Professeure adjointe
Assessment and evaluation director for the family medicine residency program
Département de médecine familiale et de médecine d'urgence
Faculté de médecine
Université Laval
miriam.lacasse@fmed.ulaval.ca

Thanks to Dr Theresa van der Goes, assessment and evaluation director at the University of British Columbia family medicine residency program, for her help with the translated version.

* This tool was validated in French only. Please contact the author if interested to conduct transcultural validation.
